# The Southernmost Known Population of the Monito Del Monte, *Dromiciops gliroides*

**DOI:** 10.1002/ece3.73227

**Published:** 2026-03-12

**Authors:** Roberto F. Nespolo, Paula Herrera, Isidora Camus, Juan Pablo Bravo, Alex D. González, Carla Toledo, Diego Vera, Francisco E. Fontúrbel, Julian F. Quintero‐Galvis

**Affiliations:** ^1^ Instituto de Ciencias Ambientales y Evolutivas Universidad Austral de Chile Valdivia Chile; ^2^ Millenium Nucleus of Patagonian Limit of Life (LiLi) Valdivia Chile; ^3^ Center of Applied Ecology and Sustainability (CAPES) Santiago Chile; ^4^ Millennium Institute for Integrative Biology (iBio) Santiago Chile; ^5^ Nature Tourism Program, Universidad Austral de Chile Coyhaique Chile; ^6^ Instituto de Biología Pontificia Universidad Católica de Valparaíso Valparaíso Chile; ^7^ Parasitic Plants Research Group Universidade Estadual do Sudoeste da Bahia Vitória da Conquista Bahia Brazil; ^8^ Departamento de Ciencias, Facultad de Artes Liberales Universidad Adolfo Ibáñez Santiago Chile

**Keywords:** conservation, *Dromiciops gliroides*, Microbiotheria, Patagonia, range extension, temperate rainforest

## Abstract

The monito del monte (genus *Dromiciops*) is a small arboreal marsupial endemic to the temperate rainforests of southern South America, and the sole extant representative of the order Microbiotheria. This lineage, considered a sister group of Australian marsupials, is of great evolutionary, ecological, and conservation significance. Until recently, the southern distribution limit of *D. gliroides* was thought to extend as far as Futaleufú in Chile (~43° S). Here, we document a new population approximately 100 km southwards, near Río Figueroa in central Patagonia (44° S). A specimen collected at Fundo La Bella Patagonia was genetically assigned to the southern clade of *D. gliroides* through mitochondrial (Cytb) and nuclear (IRBP) markers, clustering with populations from Chiloé Island and the southern Andes. This discovery extends the known range of the species into an area where *Nothofagus–Chusquea* forest persists, highlighting the resilience of this relict lineage under increasingly colder and seasonal conditions. The discovery of this new record strongly suggests the existence of dense, previously undocumented populations in the Aysén region and potentially in areas further south. Conservation efforts should, therefore, prioritize these newly documented populations, which may act as critical refugia and genetic reservoirs for Microbiotheria in the face of ongoing climate and land‐use change.

## Graphical abstract

A range extension for a relict south American marsupial.

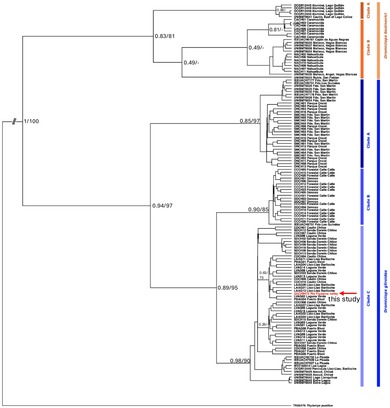

## 1 Introduction

In animal ecology, delimiting a species distribution range is key for understanding its place in nature (Sexton et al. 2009). This range, the geographic area where a species is found, is not a static outline but a dynamic reflection of a complex interplay between the species' physiological tolerances, behavioral adaptations, and biotic interactions, such as competition and predation, all constrained by historical factors and dispersal ability (Comte et al. 2024). It allows us to identify biodiversity hotspots, predict how species might respond to habitat fragmentation and climate change, and inform targeted conservation strategies by pinpointing areas of critical habitat, core populations, and range edges where species are often most vulnerable (Brown et al. 1996).

The two species of monito del monte (*Dromiciops bozinovici* and *D. gliroides* ) are small, nocturnal, arboreal marsupials endemic to the temperate forests of southern South America and the sole extant representatives of the order Microbiotheria, a lineage with deep evolutionary roots that extends back to the Paleogene (D'Elía et al. 2016; Hershkovitz 1999; Quintero‐Galvis et al. 2021). As a sister group to Australidelphia (Australian marsupials), *Dromiciops* represents a key biogeographic link between the South American and Australasian faunas, most likely established via an Antarctic land connection during the Cretaceous (Feng et al. 2022). Fossil evidence indicates that Microbiotheriids were once widespread across southern South America, from present‐day Bolivia to Antarctica, but most lineages disappeared during the Neogene, leaving Dromiciops as a relict inhabitant of the Valdivian temperate rainforest (Goin et al. 2016; Hershkovitz 1999).

The Valdivian rainforest—dominated by *Nothofagus* species and a rich assemblage of native woody vines, bamboos, and epiphytes—has served as a climatic and ecological refuge for *Dromiciops* for over 13 million years (Goin et al. 2016; Goin and Carlini 1995; Quintero‐Galvis et al. 2021). However, its distribution has contracted and fragmented repeatedly due to major geological and climatic events, including Miocene marine transgressions, Pleistocene glaciations, and, more recently, anthropogenic deforestation (Echeverria et al. 2006; Fontúrbel et al. 2022; Quintero‐Galvis et al. 2022). As a result, the current range of *Dromiciops* is best described as a mosaic of populations occupying isolated forest patches, with many areas between them poorly surveyed (Martin 2010).

For decades, *Dromiciops* was considered a monotypic genus with *D. gliroides* as the only known species, spanning a latitudinal geographic range of ~1000 km in Chile and Argentina (Hershkovitz 1999). However, the integrative taxonomic work has revealed substantial genetic and morphological structuring across its range. D'Elía et al. (2016) proposed the existence of three species—*D. bozinovici, D. mondaca*, and *D. gliroides* —each restricted to narrower distributions than previously thought. More recently, Quintero‐Galvis et al. (2021, 2022, 2024) applied genomic and coalescent‐based analyses to clarify the phylogeographic history of the group, confirming deep divergence among lineages and suggesting that isolation occurred since the Miocene (~13 Mya), which has shaped their present‐day genetic structure. These refined genomic analyses confirmed the existence of two *Dromiciops* species. *Dromiciops bozinovici*, the oldest lineage, occurring in the northern section of the range, approximately between 35° S and 39° S, whereas *D. gliroides*, the southern species, is distributed between 39° S and 43° S. According to Quintero‐Galvis et al. (2022, 2024), *D. mondaca* would be considered a subspecies (*D. gliroides mondaca*), restricted to the coastal range north of Valdivia in Chile.

Both *Dromiciops* species have ecological significance as keystone seed dispersers, particularly for the hemiparasitic mistletoes *Tristerix corymbosus* and *Desmaria mutabilis*, for which these marsupials are exclusive dispersers (Amico and Aizen 2000; Orellana et al. 2025; Pincheira et al. 2024). They also contribute to the dispersal of more than 25 other native plant species, playing a crucial role in forest regeneration and connectivity (Vazquez, Ripa, et al. 2023; Vazquez, Schenone, et al. 2023). As an arboreal species, *Dromiciops* moves with agility through the forest canopy, using the entire vertical space of the habitat and reaching heights of up to 30 m (Godoy‐Güinao et al. 2018; Mejías et al. 2022). Beyond their ecological role, *Dromiciops* exhibit remarkable adaptations to the cold environments of southern South America. They are social marsupials that often gather in groups and build spherical nests primarily from the leaves of *Chusquea quila* , intertwined with mosses and other plant fibers (Honorato et al. 2016; Nespolo et al. 2022; Vazquez et al. 2020). They also hibernate, experiencing a profound metabolic depression during 5–6 months each year, during which body temperature equals environmental temperature and physiological activity is drastically reduced (Bozinovic et al. 2004; Nespolo et al. 2021). This physiological adaptation allows them to conserve energy when food resources are scarce and climatic conditions are harsh, particularly in the cold, wet winters of southern South America (Abarzúa et al. 2023; Nespolo et al. 2024).

Despite the ecological importance of *Dromiciops*, its evolutionary singularity, and complex overwintering strategies, the distributional limits of *Dromiciops* sp. and records are constantly changing due to advances in new identification technologies, citizen science, scientific collaborations, and the discovery of new records. For instance, Mejías et al. (2021) documented the northernmost Andean population of *D. bozinovici* at 35° S and 1696 m in elevation in Chile, and Vazquez et al. (2021) expanded the known distribution of the genus almost 300 km to the north in the province of Neuquén, Argentina. While Oda et al. (2019) extended the southern distribution of *D. gliroides* by 100 km beyond the Valdivian rainforest, to Chaitén and Futaleufú (43° S) in Chile, it was also found in Parque Nacional Los Alerces, Chubut, Argentina (Amico 2024). Moreover, in recent years, numerous findings have been corroborated by photographic evidence or media coverage yet remain unpublished in the scientific literature. Here, we report one of the most significant range extensions described over the last decade, encompassing a southward extension of approximately 100 km, from the previous report at Futaleufú in Chile.

## 2 Materials and Methods

### 2.1 Study Area and Specimen Collection

**FIGURE 1:**
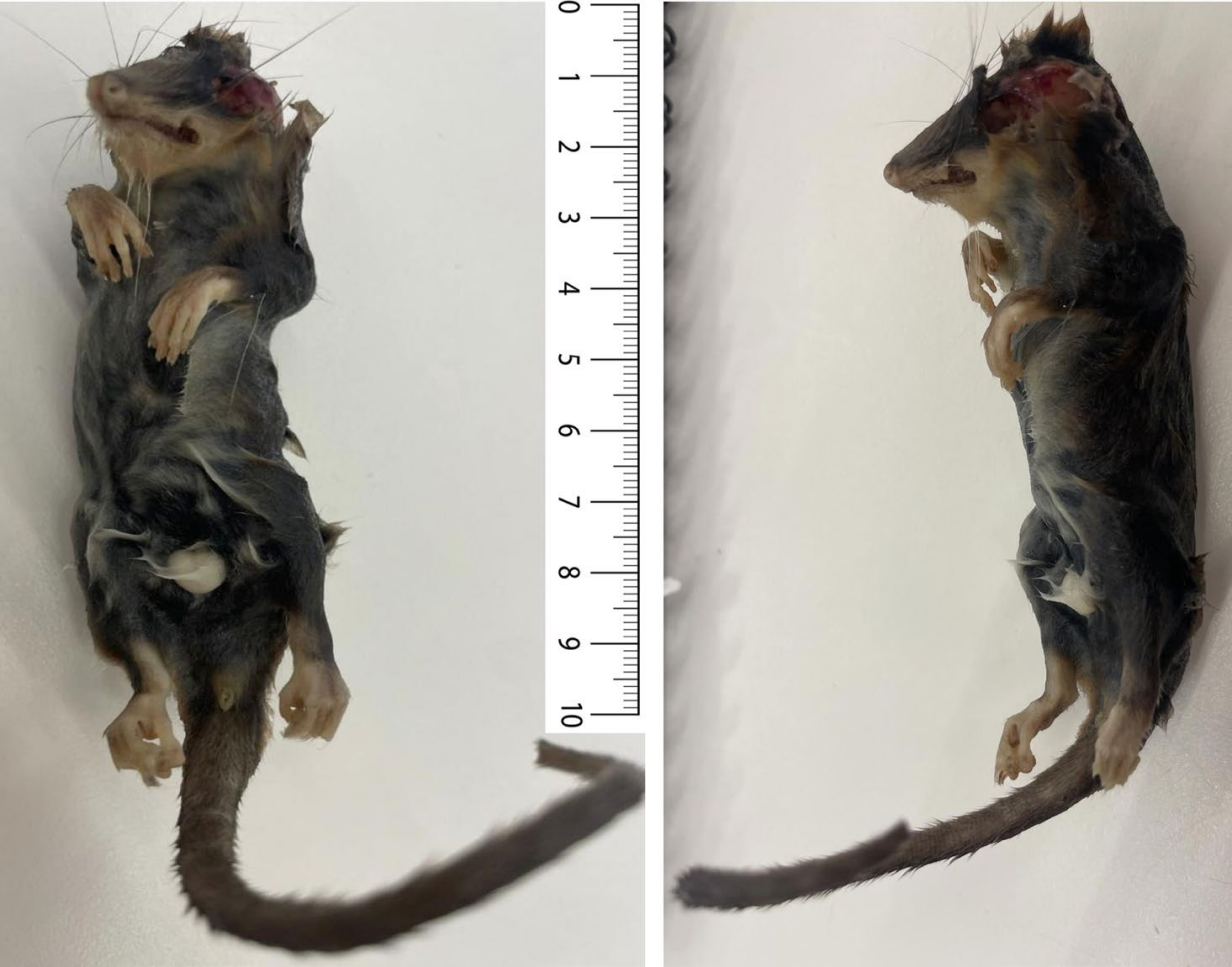
Male **s**pecimen of *Dromiciops gliroides* used in this study, found dead at Rio Figueroa apparently attacked by a predator as revealed by the head wound. The animal was preserved in 96% alcohol for 3 weeks until processed.

Field observations were conducted in March 2025 in the Río Figueroa valley, Aysén Region, central Patagonia, Chile. The flora of the Aysén Region reflects cold, humid conditions and low historical human disturbance. Vegetation is dominated by temperate evergreen and deciduous forests, particularly *Nothofagus* species such as *N. pumilio* (lenga), *N. antarctica* (ñirre), and *N. betuloides* (Magellanic coigüe), which define large portions of the landscape. In wetter areas, forests include *Tepualia stipularis*, *Drimys winteri* , and *Podocarpus nubigenus* , with dense understories of mosses, liverworts, and ferns. Extensive peatlands and wetlands support specialized hydrophytic flora, while the eastern sector transitions into Patagonian steppe vegetation adapted to strong winds and frost. A single adult male *Dromiciops* (27 g; Figure 1) was found dead on the forest floor in the vicinity of Fundo La Bella Patagonia (44°13′10.5″ S, 71°59′0.57″ W). The carcass showed signs consistent with predation by a raptor (e.g., head wounds, partially plucked fur), but remained sufficiently intact for tissue sampling. No capture or euthanasia procedures were involved. After tissue extraction, the specimen was curated and deposited in the Colección de Mamíferos, at the Universidad Austral de Chile (accession number UACH 9472). Muscle tissue was preserved in 96% ethanol at −80°C for subsequent molecular analyses.

### 2.2 DNA Extraction and Amplification

Genomic DNA was extracted from muscle tissue using the GenJet Genomic DNA Purification Kit (Thermo Fisher Scientific) following the manufacturer's protocol (product number K0721). Two loci were targeted: (i) the mitochondrial cytochrome b gene (Cytb, ~1140 bp), and (ii) the nuclear interphotoreceptor retinoid binding protein gene (IRBP, ~1200 bp). Cytb was amplified using primers TDKD (5′‐CCTGAAGTAGGAACCAGATG‐3′) and LCNTL (5′‐CACYWTYAACWCCCAAAGCT‐3′) following Himes et al. (2008). IRBP was amplified following Springer et al. (1997) using primers +IRBP217 and −IRBP1531, with alternate primers IR‐H (5′‐AACCTAATGGGGATGCAAGAAG‐3′) and IR‐I (5′‐TCCGAYCCCCAAATGCTGGCCA‐3′) used where required. PCR reactions contained 1× PCR buffer, 2.0 mM MgCl_2_, 0.2 mM dNTPs, 0.4 μM of each primer, 1 U Taq polymerase, and ~50 ng template DNA. Cycling parameters were 94°C for 2 min; 35 cycles of 94°C for 30 s, 50°C–55°C for 45 s, 72°C for 1 min; and a final extension of 72°C for 10 min. Amplicons were visualized on 1.5% agarose gels, purified with ExoSAP‐IT (Applied Biosystems), and sequenced bidirectionally on an ABI DNA Analyzer at Austral Omics, Universidad Austral de Chile.

### 2.3 Sequence Editing and Alignment

Forward and reverse reads were assembled and manually checked in Geneious v.11.1.4 (Kearse et al. 2012). Alignments were generated using MAFFT v.7 (Katoh and Standley 2013) under default parameters. To place the new sample in a phylogenetic context, 132 Cytb and IRBP sequences from *D. gliroides* and *D. bozinovici* were retrieved from GenBank (Himes et al. 2008; Quintero‐Galvis et al. 2021; Suárez‐Villota et al. 2018). The specific GenBank accession numbers for these previously published sequences are provided in the original source papers and their associated Supporting Information. Outgroups from Didelphidae (*Monodelphis* sp.) were included to root trees. All new sequences were deposited in GenBank with the accession numbers PX513671 (Cytb) and PX513672 (IRBP).

### 2.4 Phylogenetic Analyses

Maximum likelihood (ML) analyses were conducted in IQ‐TREE v1.6.8 (Nguyen et al. 2015). For the CYTB dataset, the GTR + I + G substitution model was specified, whereas the TPM3uf + I + G model was applied to the IRBP dataset. Analyses were run implementing 1000 ultrafast bootstrap replicates to assess nodal support. All other parameters were left at default settings. Bayesian inference (BI) was implemented in BEAST2 v.2.7 (Bouckaert et al. 2019, 2014) under an uncorrelated lognormal relaxed clock and Yule speciation prior, with two independent MCMC runs of 50 million generations, sampling every 5000 steps. Convergence was assessed in Tracer v.1.7, considering ESS > 200 as sufficient. Consensus trees were summarized in TreeAnnotator and visualized in FigTree v.1.4. (Rambaut and Drummond 2012).

### 2.5 Distribution Map

**FIGURE 2:**
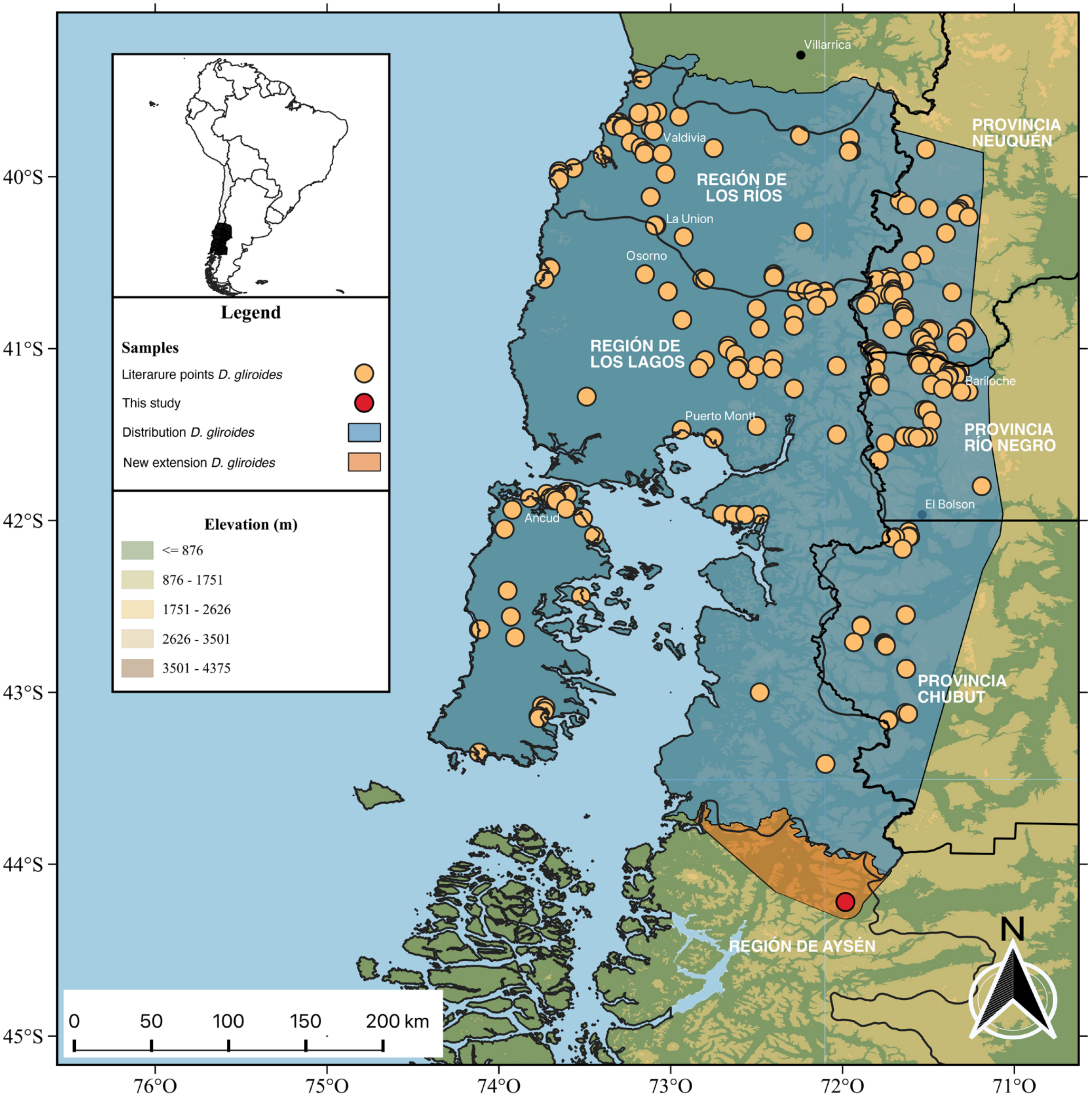
Map showing the distribution of *Dromiciops gliroides* based on previous reports (see Table 1). Red point is the new record from Río Figueroa.

A distribution map of *Dromiciops gliroides* was created based on an exhaustive review of the literature (Martin 2010; Nespolo et al. 2024; Vazquez, Ripa, et al. 2023; Vazquez, Schenone, et al. 2023) and the GBIF record. The details of each specimen and original description are provided in Table 1. A total of 390 localities were registered to map the presence of *D. gliroides* in Chile and Argentina (Figure 2). Distribution and geographic record data were compiled and visualized using a Geographic Information System using QGIS 3.40 software (Graser et al. 2025). Genetic group identifiers were employed to classify and differentiate occurrence records. Additionally, a new record was added to the database and highlighted in red on the map for easy identification and distinction from previously reported records.

**TABLE 1:** Geographical information for each individual sample used in the phylogenetic analysis.

Locality	Latitude	Longitude	ID	Original description
Ñuble, San Fabian	−36.5833	−71.4667	UWBM78633	Himes et al. (2008)
Caramavida	−37.6960	−73.1969	CACH01, CACH02, CACH03, CACH04, CACH05, CACH06, CACH07	Quintero‐Galvis et al. (2021)
Malleco, Curacautín, Termas de Tolhuaca	−37.8245	−72.9712	UWBM78636, UWBM78637, UWBM78638	Himes et al. (2008)
Malleco, Angol, Vegas Blancas	−37.8333	−72.8667	UWBM78635, UWBM78634	Himes et al. (2008)
Nahuelbuta	−38.2333	−71.7333	NACH02, NACH03, NACH04, NACH06, NACH07, NACH08, NACH09, NACH10, NACH11	Quintero‐Galvis et al. (2021)
Cajón de Aguas Negras	−38.5333	−71.1833	IEEUACH6161	Himes et al. (2008)
Cautín, Cunco, East of Lago Colico	−39.1000	−71.8667	IEEUACH616	Himes et al. (2008)
Aluminé, Lago Quillén	−39.3667	−71.2333	OCGR12450, OCGR12449, OCGR12446, OCGR12445	Himes et al. (2008)
Fdo Los Guindos	−39.6288	−73.1870	IEEUACH5731	Himes et al. (2008)
Fdo. San Martín	−39.6288	−73.1870	UWBM78624, UWBM78626, UWBM78628, UWBM78629, UWBM78630, UWBM78631, IEEUACH7178, IEEUACH7177	Suárez‐Villota et al. (2018)
Fdo. San Martín	−39.6500	−73.1941	SMCH01, SMCH02, SMCH03, SMCH04, SMCH05, SMCH06, SMCH07, SMCH08, SMCH42, SMCH43, SMCH44	Quintero‐Galvis et al. (2021)
Parque Oncol	−39.7027	−73.3263	ONCH01, ONCH02, ONCH03, ONCH04, ONCH06, ONCH08, ONCH09, ONCH10, ONCH11, ONCH12, ONCH13, ONCH16	Quintero‐Galvis et al. (2021)
Donoso	−39.8658	−73.1541	DDCH01, DDCH03, DDCH04, DDCH05, DDCH06, DDCH07	Quintero‐Galvis et al. (2021)
Forestal Calle Calle	−39.8667	−73.0500	CCCH03, CCCH10, CCCH11, CCCH12, CCCH13, CCCH14, CCCH15, CCCH20, CCCH21, CCCH22, CCCH23, CCCH24, CCCH26	Quintero‐Galvis et al. (2021)
Entre Lagos	−40.6667	−72.2667	UWBM78644, UWBM78645	Himes et al. (2008)
Los Lagos, Estancia Paso Coihue	−40.8833	−71.2833	MVZ184914	Himes et al. (2008)
Llao‐Llao Bariloche	−41.0333	−71.5333	LAAG01, LAAG02, LAAG04, LAAG07, LAAG09, LAAG11, LAAG12	Quintero‐Galvis et al. (2021)
Llanquihue, East of Lago Llanquihue	−41.1833	−72.5500	UWBM78642	Himes et al. (2008)
La Picada	−41.3574	−71.5103	IEEUACH7028, IEEUACH7027, IEEUACH5732	Himes et al. (2008)
Laguna Verde	−41.3574	−71.5103	LVAG01, LVAG02, LVAG03, LVAG04, LVAG05, LVAG06, LVAG08, LVAG09, LVAG10, LVAG11, LVAG12, LVAG13, LVAG14, LVAG15	Quintero‐Galvis et al. (2021)
Puerto Blest	−41.3574	−71.5103	PBAG01, PBAG02, PBAG03, PBAG04, PBAG06	Quintero‐Galvis et al. (2021)
Peninsula Llao‐Llao, Bariloche	−41.5197	−71.5625	OCGR12440	Himes et al. (2008)
Caulin Chiloe	−41.8333	−73.6090	CDCH01, CDCH04, CDCH05, CDCH07, CDCH08, CDCH09, CDCH10	Quintero‐Galvis et al. (2021)
Senda Darwin Chiloe	−41.8691	−73.6830	SDCH02, SDCH03, SDCH04, SDCH07, SDCH08, SDCH09, SDCH12, SDCH13, SDCH15, SDCH16	Quintero‐Galvis et al. (2021)
Ancud, Chiloé	−41.8833	−73.6833	UWBM79639, UWBM79629	Himes et al. (2008)
Río Figueroa valley	−44.2196	−71.9835	UACH9472	This study

## 3 Results

**FIGURE 3:**
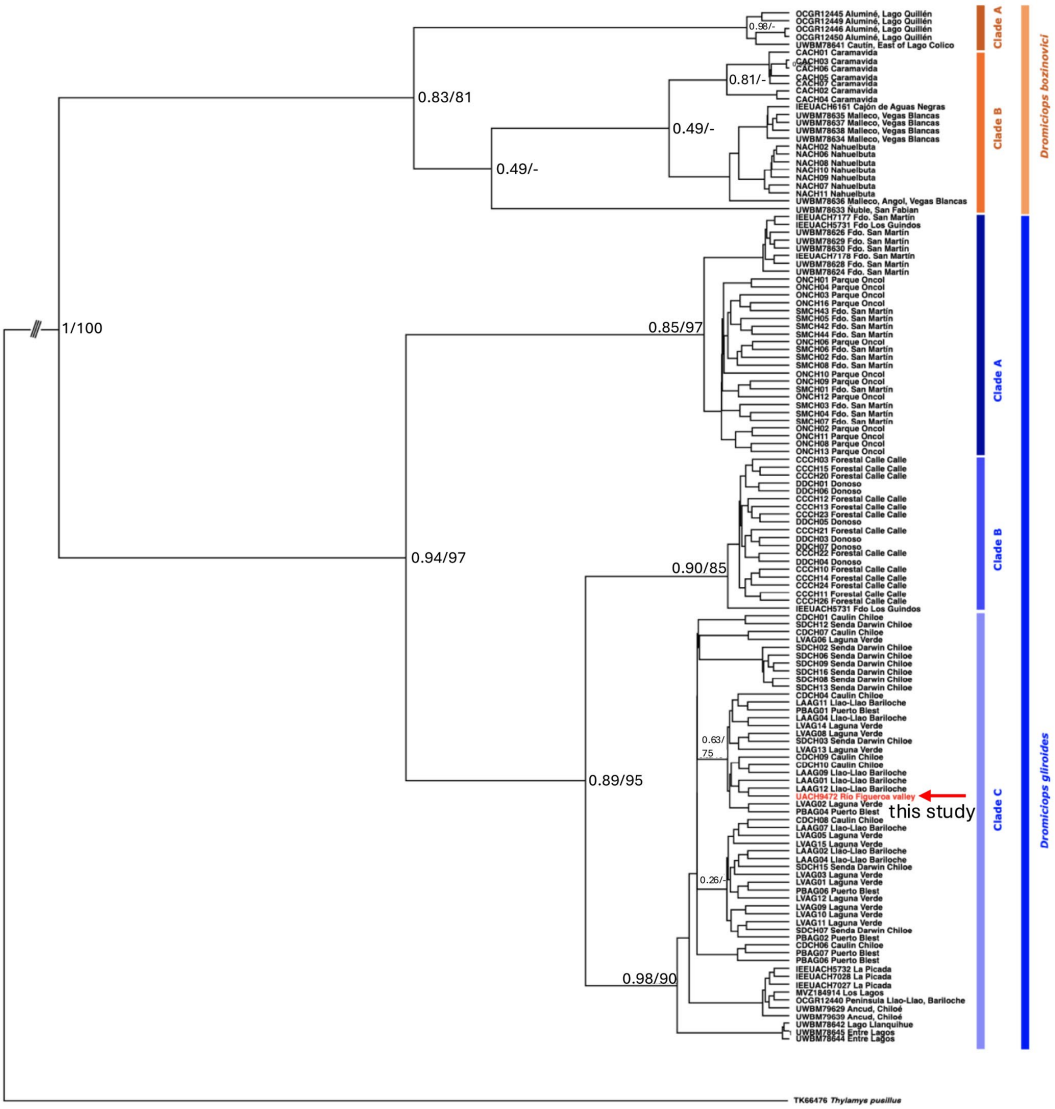
Maximum clade credibility tree inferred from IB (Cytb + IRBP). Values in nodes correspond to the posterior probability (first number)/bootstrap (second number) support values obtained in the Bayesian inference and Maximum likelihood analysis. Indicated by red and arrow, is the new specimen. Each tip of the phylogeny include the specimen ID and the locality.

The new presence records reported here represent a range extension for *Dromiciops* of ~100 km south of Futaleufú (its previous southern limit), now reaching Río Figueroa, being the first occurrence record of *D. gliroides* in the Aysén Region in Chile (Figure 3). The updated distribution range integrates literature records and museum specimens, demonstrating a marked range extension into central Patagonia, a largely neglected region in terms of field sampling. The range extension comprises continuous *Nothofagus–Chusquea* forest stands, which are suitable habitats, consistent with other known *Dromiciops* populations.

Both Maximum Likelihood (ML) and Bayesian Inference (BI) reconstructions yielded congruent topologies, clearly separating the *D. bozinovici* lineage from the *D. gliroides* lineages. Phylogenetic analyses of mitochondrial (Cytb) and nuclear (IRBP) markers unequivocally placed the specimen collected at Río Figueroa within *Dromiciops gliroides* (Figure 3). Within *D. gliroides* , the new specimen nested within clade C, which encompasses the southern portion of the distribution. The Río Figueroa sequence is grouped within *D. gliroides* clade C, clustering with high nodal support (posterior probability = 0.98, bootstrap = 90) alongside sequences derived from Chiloé Island and the southern Andean populations in Chile and Argentina. No topological conflicts were observed between mitochondrial and nuclear partitions, and both markers consistently supported the genetic assignment to clade C of *D. gliroides* .

## 4 Discussion

The extension of the distribution range of *Dromiciops gliroides* roughly 100 km south of its previously documented distribution boundary significantly reshapes our understanding of the ecology and biogeography of this unique marsupial. Its distribution has long been tied to the temperate broadleaf rainforests of southern South America (sensu Woodward et al. 2004), where it plays a critical ecological role as a keystone seed disperser (Amico and Aizen 2000; Fontúrbel et al. 2022). Recording *Dromiciops* individuals is challenging, requiring modified tomahawk traps, specialized bait, and a carefully planned design (e.g., Mejías et al. 2021). Finding an individual *D. gliroides* and recording its origin is important for its conservation and contributes to sites with records of conserved environments where the species has not been studied, and that could serve as refuges. Extending its known range toward Río Figueroa, at latitude 44° S (central Patagonia), our findings offer a glimpse into the resilience of this relict lineage and raise interesting questions about the continuity of suitable habitats further south into Patagonia (Nespolo et al. 2024).

From a biogeographic perspective, this finding highlights the persistence of Gondwanan assemblages in Patagonia. Fossil evidence and ecological associations strongly link *Dromiciops* with the Nothofagus–Chusquea forest complex, which extends deep into southern Chile and Argentina (Hershkovitz 1999). This new southern record suggests that these forests continue to provide the structural and climatic conditions necessary for the survival of *Dromiciops*, including dense arboreal substrates, fleshy‐fruited plants, and the cool winters required for its hibernation strategy (Nespolo et al. 2024). Physiologically, *D. gliroides* exhibits an extraordinary capacity for multi‐day torpor, reducing energy expenditure by more than 90% during the austral winter (Abarzúa et al. 2023; Nespolo et al. 2024). Such adaptations may facilitate its persistence in increasingly colder and seasonal environments toward higher latitudes.

Ecologically, the presence of *D. gliroides* in this region reinforces its role as a keystone species in Patagonian forests. The species disperses seeds of numerous fleshy‐fruited plants (Amico and Nickrent 2009), most notably the mistletoes *Tristerix corymbosus* and *Desmaria mutabilis* (Amico and Aizen 2000; Fontúrbel and Orellana 2022; Pincheira et al. 2024), which are considered foundational elements for forest community dynamics (Olivares et al. 2025). Extending its known distribution southward implies that these ecological processes may also be active in previously unexplored parts of Patagonia.

Conservation implications are substantial (both *Dromiciops* species are categorized as near threatened by the UICN). While this discovery broadens the area of occupancy of *D. gliroides*, it also places new emphasis on the need to protect southern Patagonian forests, which are increasingly threatened by deforestation, infrastructure development, and climate‐driven vegetation changes (Soler et al. 2022; Veblen et al. 2011). Northern populations, particularly those of *D. bozinovici*, face pressures from habitat fragmentation and warming winters, making the southern refugia of *D. gliroides* potentially critical for the long‐term persistence of the lineage (Nespolo et al. 2024; Quintero‐Galvis et al. 2024). Conservation strategies must therefore consider these southern populations as genetic and ecological reservoirs essential for the future of Microbiotheria. Indeed, the occurrence at ~44° S raises the possibility that *D. gliroides* may extend even further south, potentially into the fjord landscapes of Aysén and perhaps as far as the northern margins of the Southern Patagonian Ice Field. The continuity of *Nothofagus* forests in these regions, combined with the physiological flexibility of *D. gliroides* , makes such a distribution plausible, as indicated by the potential distribution of *D. gliroides* (Rodríguez‐Souilla et al. 2025). A logical next step to refine this hypothesis would be habitat suitability modeling (e.g., MaxEnt or similar approaches), which could generate explicit predictions of southern range limits, guide targeted field surveys, and identify potential climatic refugia. Confirming this hypothesis will require systematic surveys using camera traps, live‐trapping, environmental DNA, and genetic analysis to detect potential isolated refugia. If verified, such findings would redefine *Dromiciops* biogeographic limits and strengthen the recognition of Patagonia as a critical stronghold for Gondwanan relict biodiversity.

In conclusion, the discovery of this new southern population of *Dromiciops gliroides* aligns with its unique evolutionary status, ecological role, and physiological specializations, highlighting the resilience of this marsupial lineage. It reinforces the idea that Patagonian temperate rainforests function as refugia for ancient biotas and underscores the urgency of conserving these habitats. Although only a single individual was detected—an obvious limitation—this record robustly documents a range extension. Given that *Dromiciops* is a social species that typically occurs at relatively high local densities, such detections are unlikely to represent truly isolated individuals; however, stronger inferences about population structure and connectivity will require additional, systematic sampling.

Future research should clarify whether these populations are demographically connected to central ones or represent isolated evolutionary lineages, knowledge essential for predicting the resilience of *Dromiciops* under accelerating global change.

## Author Contributions

**Diego Vera:** methodology (equal). **Francisco E. Fontúrbel:** conceptualization (equal), supervision (equal), writing – original draft (equal), writing – review and editing (equal). **Julian F. Quintero‐Galvis:** conceptualization (equal), data curation (equal), formal analysis (equal), methodology (equal), visualization (equal), writing – original draft (equal), writing – review and editing (equal). **Paula Herrera:** investigation (equal), methodology (equal). **Carla Toledo:** methodology (equal). **Isidora Camus:** data curation (equal), investigation (equal), methodology (equal). **Alex D. González:** methodology (equal). **Juan Pablo Bravo:** investigation (equal), methodology (equal). **Roberto F. Nespolo:** conceptualization (equal), methodology (equal), project administration (equal), resources (equal), supervision (equal), writing – original draft (equal), writing – review and editing (equal).

## Funding

This work was supported by the Fondecyt 1221073, 1250935, 11260582.

## Ethics Statement

The authors have nothing to report.

## Conflicts of Interest

The authors declare no conflicts of interest.

## Data Availability

The authors have nothing to report.
